# Characterization of the complete chloroplast genome sequence of the medicinal plant *Mirabilis himalaica*

**DOI:** 10.1080/23802359.2020.1788449

**Published:** 2020-07-14

**Authors:** Fang Yuan, Yuanjiang Xu, Kaihui Zhao, Yazhou Lu, Xiaozhong Lan

**Affiliations:** TAAHC-SWU Medicinal Plant Joint R&D Center, Tibetan Collaborative Innovation Center of Agricultural and Animal Husbandry Resources, Food Science College, Tibet Agriculture & Animal Husbandry University, Nyingchi, Tibet, China

**Keywords:** *Mirabilis himalaica*, Endangered species, chloroplast genome, conservation

## Abstract

*Mirabilis himalaica* is an old and popular medicinal plant used in traditional Tibetan folk medicine. Here, we reported the complete chloroplast genome sequence of *Mirabilis himalaica.* The assembled chloroplast genome was 154,348 bp long, containing a large single-copy region of 85,809 bp, a small single-copy region of 17,935 bp, and a pair of inverted repeat regions of 25,302 bp. It had 36% GC content and encoded 131 genes including 86 protein-coding genes, eight rRNA genes, and 37 tRNA. Fifteen and two genes contained one and two introns, respectively. Phylogenetic analysis revealed that *Mirabilis himalaica* was sister to *Nyctaginia capitata.*

## Introduction

*Mirabilis himalaica* (Edgew.) Heimerl (Nyctaginaceae) is an important medicinal plant widely used in traditional Tibetan folk medicine for the treatment of kidney diseases, edema, periorbital puffiness, low back pain, arthralgia, inflammation, etc. (Zhang et al. 1997; Yang et al. 2012). The species is endemic to the Tibet, China and its artificial domestication and cultivation are in the initial development stage (Guo et al. 2014). Long-term exploitation of wild *Mirabilis himalaica* resources coupled with an increasing market demand is threatening the survival of the species (IUCN 2003). Therefore, to conserve this precious species and facilitate breeding studies, it is essential to clarify its systematic position and generate molecular tools. Herein, we sequenced and assembled the complete chloroplast genome of *Mirabilis himalaica* (Genbank accession number: MT535664) and performed a phylogenetic analysis with 34 other species.

Genomic DNA was obtained from fresh leaves of *Mirabilis himalaica* collected from Nyingchi City (N: 29°40′23.40″, E: 94°20′28.28″), Tibet Autonomous Region, China in August 2019 using the CTAB method (Doyle and Doyle 1987). The voucher specimen was deposited at Tibet Agriculture and Animal Husbandry University (Accession number: ZY19082301). The chloroplast genome sequencing was performed at Wuhan bio-mall biotechnology Co., Ltd, Wuhan, China, using the Illumina Hiseq sequencing technology (Illumina, San Diego, CA, USA).

The generated 150 bp paired-end reads were quality-checked using the Fastqc tool (http://www.bioinformatics.babraham.ac.uk/projects/fastqc/) and low-quality sequences were filtered out. Then, the chloroplast genome was reconstructed using SPAdes v3.9.0 (Nurk et al. 2013) and MITObim v1.8 (Hahn et al. 2013). The assembled chloroplast genome was annotated using CpGAVAS (Liu et al. 2012). The complete chloroplast genome of *Mirabilis himalaica* was a circular DNA molecule of 154,348 bp in length, representing an average sequencing depth of 7,853.2X. The chloroplast genome had overall 36% GC content and encoded 131 genes including 86 protein-coding genes, 8 rRNA genes, and 37 tRNA. It showed a typical quadripartite structure consisting of two reverse repeated regions (IRa and IRb) of 25,302 bp in length, separated by a large single-copy region (LSC: 85,809 bp) and a small single-copy region (SSC: 17,935 bp). The genes *trnK-UUU*, *rps16*, *trnG-UCC*, *atpF*, *rpoC1*, *trnL*-*UAA*, *trnV*-*UAC*, *petB*, *petD*, *rpl16*, *rpl2*, *ndhB*, *trnI-GAU*, *trnA-UGC*, *ndhA* contained each one intron while the genes *clpP*, *ycf3* contained two introns. The gene *rps12* was trans-spliced.

**Figure 1.**
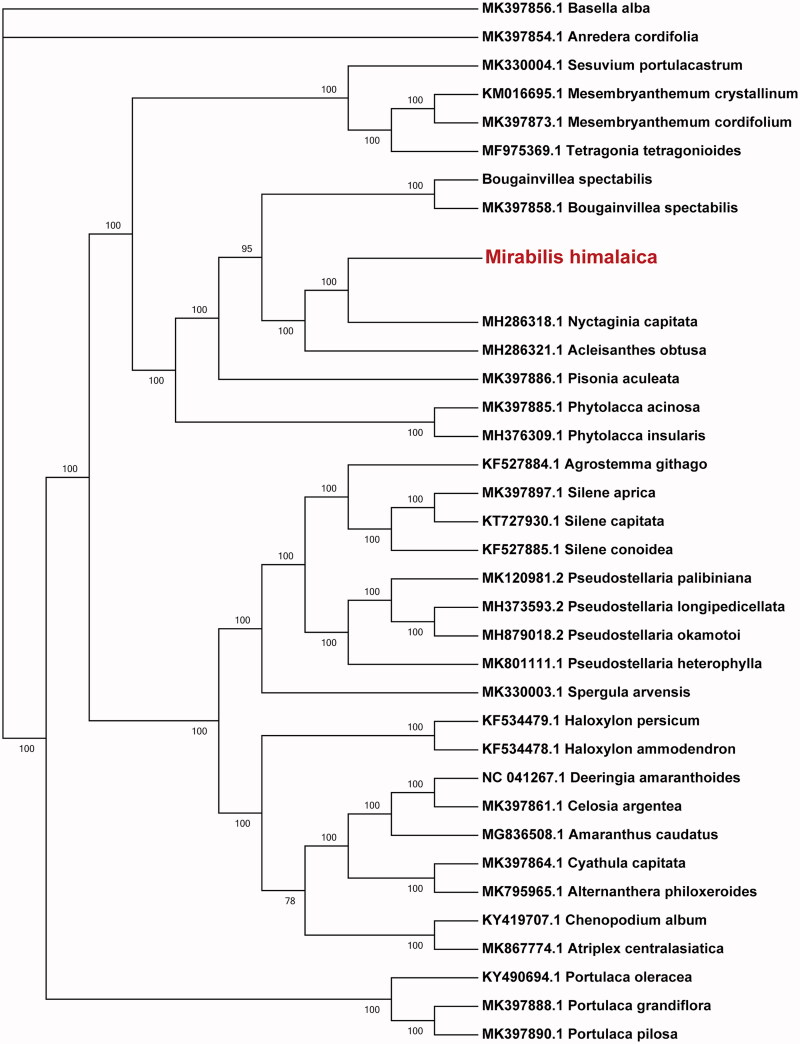
Maximum likelihood (ML) tree based on the chloroplast genome sequences from 35 species. Values along branches correspond to ML bootstrap percentages.

To ascertain the phylogenetic position of *Mirabilis himalaica* in the Nyctaginaceae family, we retrieved chloroplast genome sequences of 34 species including those from Nyctaginaceae and other families. A Maximum Likelihood phylogenetic tree was constructed with 1000 bootstraps in the program MEGA7 (Kumar et al. 2016). The result showed that *Mirabilis himalaica* was closely clustered with *Nyctaginia capitata* (Nyctaginaceae) (Figure 1).

## Data Availability

The data that support the findings of this study are openly available in GenBank of NCBI at https://www.ncbi.nlm.nih.gov/nuccore, reference number MT535664.
